# Savolitinib: A Promising Targeting Agent for Cancer

**DOI:** 10.3390/cancers15194708

**Published:** 2023-09-25

**Authors:** Tae Seung Lee, Jun Yeol Kim, Myeong Hwan Lee, In Rae Cho, Woo Hyun Paik, Ji Kon Ryu, Yong-Tae Kim, Sang Hyub Lee

**Affiliations:** Department of Internal Medicine and Liver Research Institute, Seoul National University Hospital, Seoul National University College of Medicine, Seoul 03080, Republic of Korea; rhytksa@snu.ac.kr (T.S.L.); kimjun16@snu.ac.kr (J.Y.K.); dlaudghks01@snu.ac.kr (M.H.L.); inrae0428@snu.ac.kr (I.R.C.); iatrus@snu.ac.kr (W.H.P.); jkryu@snu.ac.kr (J.K.R.); yongtkim@snu.ac.kr (Y.-T.K.)

**Keywords:** savolitinib, MET-TKI, NSCLC, gastric cancer, renal cell carcinoma, pancreatic cancer

## Abstract

**Simple Summary:**

Savolitinib is a highly specific inhibitor of the MET tyrosine kinase. Both preclinical and clinical studies have shown its potential as a treatment for various cancers, including non-small cell lung cancer (NSCLC) as well as breast, head and neck, colorectal, gastric, pancreatic, and other gastrointestinal cancers. It can be used as a standalone treatment for NSCLC patients with MET mutations and in combination with EGFR inhibitors for those who have developed resistance. Moreover, it is being investigated as a neoadjuvant therapy. Furthermore, savolitinib has demonstrated efficacy in gastric cancer and may be effective in combination therapy. Additionally, it has shown effectiveness in treating renal cancer and other gastrointestinal cancers.

**Abstract:**

Savolitinib is a highly selective small molecule inhibitor of the mesenchymal epithelial transition factor (MET) tyrosine kinase, primarily developed for the treatment of non-small cell lung cancer (NSCLC) with *MET* mutations. It is also being investigated as a treatment for breast, head and neck, colorectal, gastric, pancreatic, and other gastrointestinal cancers. In both preclinical and clinical studies, it has demonstrated efficacy in lung, kidney, and stomach cancers. Savolitinib is an oral anti-cancer medication taken as a 600 mg dose once daily. It can be used as a monotherapy in patients with non-small cell lung cancer with *MET* mutations and in combination with epidermal growth factor receptor (EGFR) inhibitors for patients who have developed resistance to them. Furthermore, savolitinib has shown positive results in gastric cancer treatment, particularly in combination with docetaxel. As a result, this review aims to validate its efficacy in NSCLC and suggests its potential application in other gastrointestinal cancers, such as pancreatic cancer, based on related research in gastric and renal cancer.

## 1. Introduction

Cancer continues to be a major cause of death worldwide. Recently, large-scale genetic profiling in cancer has been utilized to identify additional therapeutic agents through genetic profiling [1]. Consequently, prospective molecular profiling has emerged as an essential instrument in the diagnosis and treatment of cancer. Genomic alterations of the *MET* proto-oncogene receptor tyrosine kinase have been well studied in non-small cell lung cancer (NSCLC). Mesenchymal epithelial transition factor (MET) tyrosine kinase inhibitors (TKIs) such as crizotinib, capmatinib, tepotinib, savolitinib, and cabozantinib, which target *MET* exon 14 mutations, have been approved by the Food and Drug Administration (FDA). These FDA-approved MET TKIs are widely used as therapeutic agents worldwide [2,3].

MET TKIs are classified into three types based on their mechanism of action. Type I MET TKIs act as adenosine triphosphate (ATP) competitors and target the ATP binding pocket of the active form of the MET receptor. There are two subtypes of Type I inhibitors: Ia inhibitors, such as crizotinib, which target the amino acid residue known as G1163, and Ib inhibitors, such as capmatinib, tepotinib, and savolitinib, which strongly bind to the amino acid residue Y1230. Type II MET TKIs, such as cabozantinib, are also ATP competitors but bind to the inactive state of the MET receptor, inhibiting ATP-dependent activation by targeting the receptor’s inactive form. Type III TKIs are allosteric inhibitors [4]. We will focus on discussing savolitinib, which belongs to the Type Ib MET TKI class. Savolitinib specifically interacts with the Y1230 residue and effectively inhibits the activity of the MET receptor. By targeting this specific region, savolitinib successfully hinders the signaling pathway associated with MET activation.

## 2. Hepatocyte Growth Factor-Mesenchymal Epithelial Transition Factor Signaling

### 2.1. Overview of HGF-MET Signaling

Mesenchymal epithelial transition factor (MET), also known as c-MET or the Hepatocyte Growth Factor (HGF) receptor, is a tyrosine kinase receptor that is typically expressed in various cell types, including epithelial cells, endothelial cells, neurons, hepatocytes, and hematopoietic cells [1,5,6,7,8]. MET, along with its ligand HGF, plays a crucial role in multiple cellular processes, including cell proliferation, motility, morphogenesis, angiogenesis, tissue regeneration, and the transition from epithelial to mesenchymal cells. It is also involved in wound healing, normal liver development, embryonic placental development, and the development of neurons and muscles [9,10,11].

HGF is the ligand for MET, and its binding induces the dimerization of MET receptors and the subsequent phosphorylation of specific tyrosine residues, called Tyr1230, Tyr1234, and Tyr1235, within the kinase domain. This phosphorylation creates a docking site for proteins involved in RTK-mediated signal transduction, leading to the activation of downstream signaling pathways [12,13]. One of the downstream signaling pathways activated by MET involves the attachment of phosphorylated GAB1, which further recruits docking molecules and enzymes, including PI3K, CRK-like protein (CRKL), and SRC homology 2 domain-containing phosphatase 2 (SHP2). These activated signaling pathways include phosphoinositide 3-kinase (PI3K)/AKT (protein kinase B), mitogen-activated protein kinase (MAPK), and NF-kB [14,15,16].

HGF-bound MET receptors are normally ubiquitinated, and they are internalized via endocytosis, where they are either degraded or recycled back to the plasma membrane. Consequently, dysregulation of the MET signaling system is associated with various malignancies. Aberrant receptor trafficking, degradation, or disrupted recycling can result in sustained signaling, contributing to cellular transformation, oncogenesis, and metastasis.

### 2.2. MET Mutation

Abnormalities in the MET signaling system can arise from different mechanisms, including gene amplification, receptor protein overexpression, MET exon 14 junctional membrane skipping mutations (METex14), and MET gene chromosomal fusions [17]. The association between MET mutations and cancer development was initially observed in hereditary and sporadic forms of papillary renal cell carcinoma. There were somatic missense mutations in the tyrosine kinase (TK) domain of the MET gene. These mutations are typically gain-of-function mutations [18].

**Figure 1:**
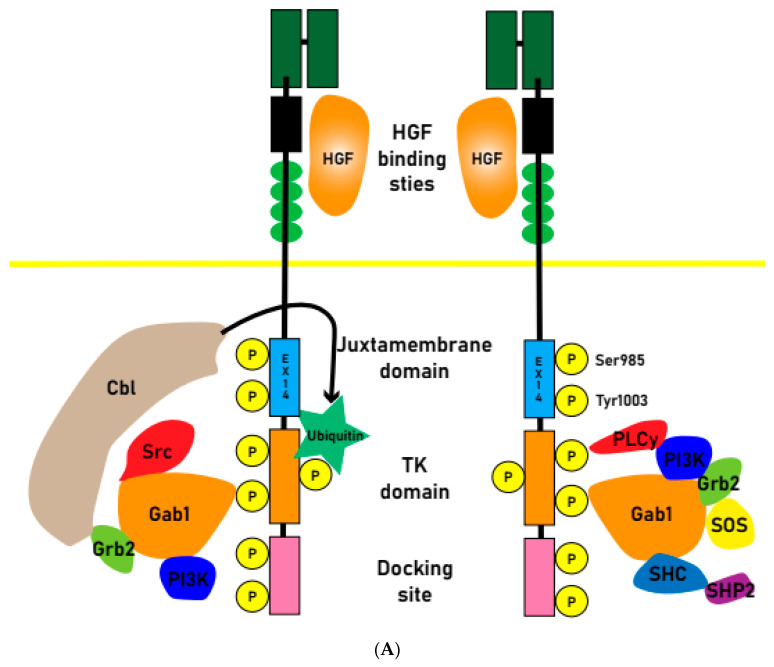

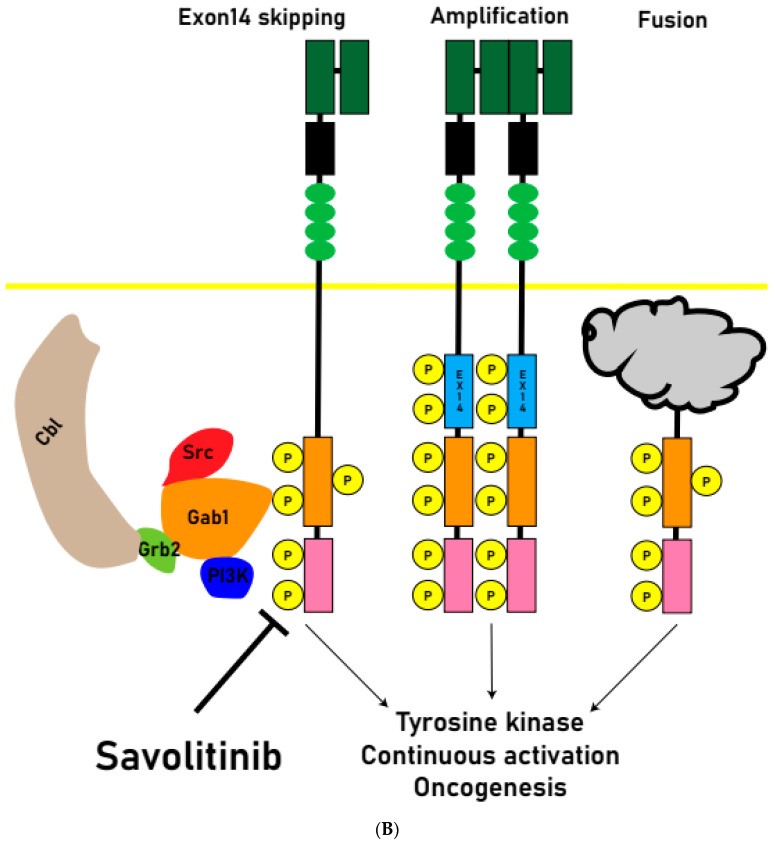
(**A**) Normal physiologic MET receptors form dimers upon HGF signaling, and Cbl binding leads to ubiquitination. Upon ubiquitination, the MET receptor is degraded, and signal transduction ceases. (**B**) Oncogenesis pathway: when exon 14 is skipped and Cbl fails to bind to its receptor and is not ubiquitinated, or when the signaling system is amplified by *MET* amplification or chromosome fusion, it continuously activates tyrosine kinases and causes oncogenesis. Savolitinib inhibits this MET signaling pathway.

Juxtamembrane domain (JMD)-deleted MET was generated by MET exon 14 skipping mutations due to intronic mutations. The expression of MET-exon14 variants in cells disrupts the association with the CBL E3 ubiquitin ligase, reducing ubiquitination and prolonging the activation of signaling molecules. The phosphorylation of MET-Y1003 in the juxtamembrane domain is typically involved in CBL binding for ubiquitination. Therefore, MET-exon14 variants may have increased protein stability and enhanced signal transduction, contributing to oncogenesis [19,20] (Figure 1A).

Abnormal activation of the MET pathway in tumor tissue, including MET gene overexpression, gene amplification, exon14 skipping, and other activating mutations (Figure 1B), is associated with shorter survival and a poor prognosis. MET amplification (METAmp) and overexpression are frequently observed genomic abnormalities in solid tumors, occurring in advanced stages of tumorigenesis and exacerbating the malignant properties of transformed cells. MET participates in crucial cellular processes such as proliferation, differentiation, and the formation of distant metastases [21].

MET exon14 skipping has been recognized as a true oncogenic driver, and MET TKIs targeting MET exon14 skipping mutations have shown significant improvements in clinical outcomes, including response rates and progression-free survival. Studies investigating the HGF/MET pathway have been conducted in various cancer types, including NSCLC, breast cancer, head and neck cancer, colorectal cancer, gastric cancer, pancreatic cancer, and other gastrointestinal cancers. Genetic alterations in the MET gene and pathway are common in solid tumors [21].

MET overexpression is indeed believed to be one of the early dysregulations in the process of carcinogenesis. In the presence of hypoxia and inflammation, MET receptors are transcriptionally upregulated, leading to their overexpression. This overexpression contributes to tumorigenesis by promoting cell proliferation, inhibiting apoptosis, and enhancing cell migration. MET overexpression has been observed in various types of cancer, including epithelial, mesenchymal, and hematologic cancers. Additionally, MET can also be overexpressed in cancers with an activated genomic signature, including those with primary and/or secondary MET amplifications or MET exon 14 junctional membrane skipping mutations.

### 2.3. HGF/MET Signaling Pathway Is Involved in Oncogenesis

During the characterization of HGF as a fibroblast-secreted protein that promotes motility and matrix invasion of epithelial cells, the induction of invasiveness into collagen by HGF was observed. HGF, a factor derived from fibroblasts, plays a role in facilitating the aggressive invasion of cancer cells. The microenvironment of metastatic tumors plays a crucial role in metastatic colonization and growth. Various stromal cells, including inflammatory cells, endothelial cells, and fibroblasts, contribute to the formation of the metastatic microenvironment. The function of HGF as a stromal cell-derived factor influences cancer cell invasiveness within the tumor microenvironment. Inhibiting HGF activity has been shown to prevent invasion induced by stromal fibroblasts.

Additionally, MET receptors present in exosomes have been found to promote the formation of a metastatic microenvironment in metastatic melanoma [22]. Exosomes derived from highly metastatic mouse and human melanoma cells contain high levels of MET receptors. These circulating exosomes localize to metastatic tissue sites and increase vascular permeability, thereby promoting tumor cell migration. Moreover, exosomes contribute to the activation of MET in bone marrow-derived cells, leading to their reprogramming into an angiogenic phenotype. These bone marrow-derived cells migrate to the lungs and can contribute to angiogenesis, invasion, and metastasis. The administration of exosomes with prominent levels of MET receptors promotes metastasis of melanoma cells that originally had low metastatic capacity [23].

## 3. Savolitinib: A Promising Targeting Agent

### 3.1. Introducing Savolitinib

Savolitinib is an oral medication classified as a selective MET inhibitor. It has been specifically developed for the treatment of several types of cancer, including NSCLC, breast cancer, head and neck cancer, colorectal cancer, gastric cancer, pancreatic cancer, and other gastrointestinal cancers. Preclinical studies have shown that savolitinib exhibits superior efficacy in these cancer types [24].

### 3.2. Metabolism

Savolitinib is rapidly absorbed. Absolute oral bioavailability was 69%, the median maximum observed concentration was 3.5 h, and the mean terminal half-life was 6.1 h. 56% were found in urine and 38% in feces. Approximately 3% of the administered dose was excreted as unmetabolized savolitinib in the urine. Savolitinib has moderate tissue distribution, low to moderate clearance, and low accumulation. Most of the elimination of savolitinib occurs through metabolism via multiple pathways. Hepatic oxidative metabolism, followed by urinary and biliary excretion, were the major elimination pathways. The concentration at which half of the maximal Hs746t tumor reduction by savolitinib was achieved and the IC50 for MET inhibition were equal to 12.5 and 3.7 nM (free drug), respectively [25]. There was a drug-drug interaction reported in which co-administration of rifampicin significantly reduced exposure to savolitinib compared to savolitinib alone [26].

### 3.3. Side Effects and Safety

Savolitinib demonstrated a well-tolerated safety profile consistent with previous clinical trials. Most adverse events were of grade 1 or 2 and resolved with dose adjustment or discontinuation. The most common treatment-related adverse events (TRAEs) were peripheral edema (56%), nausea (46%), and aminotransferase elevations (38%). The highest treatment-related grade 3 adverse event was AST elevation (13%) [27]. Common serious adverse events (SAEs) reported included hepatic dysfunction (4.3%), drug hypersensitivity reactions (2.9%), and pyrexia (2.9%). There was one treatment-related fatal SAE reported, specifically tumor lysis syndrome. It is worth noting that pulmonary interstitial pneumonia and interstitial lung disease (ILD) did not occur with savolitinib, while ILD was observed with other drugs such as tepotinib and capmatinib.

Savolitinib had a well-tolerated safety profile consistent with previous clinical trials, with most adverse events being grade 1–2 and resolved with dose adjustment and discontinuation. The most common treatment-related adverse events (TRAEs) reported were hepatic dysfunction (4.3%), drug hypersensitivity reactions (2.9%), and pyrexia (2.9%). A treatment-related fatal SAE, tumor lysis syndrome, was reported in one patient. Pulmonary interstitial pneumonia and interstitial lung disease (ILD) did not occur with savolitinib, whereas ILD was observed with tepotinib and capmatinib.

Savolitinib has been associated with the side effect of QTc prolongation, which refers to a lengthening of the QT interval on an electrocardiogram (ECG). In a study specifically examining QT/QTc, a single dose of 600 mg of savolitinib resulted in the highest root mean square ΔΔQTcF (change in QTc interval corrected for heart rate) of 12 milliseconds, observed 5 h after administration. However, savolitinib did not have any significant effects on other ECG intervals such as PR, QRS, QT, or RR intervals. The study found that QTcF prolongation occurred with a single dose of 600 mg of savolitinib, and a lesser increase of 5 milliseconds was noted with a dose of 300 mg. As a result, ongoing and future clinical trials involving savolitinib will include ECG monitoring to assess the clinical relevance of these observed QT changes [28,29]. One case reported septic shock-like symptoms when savolitinib was used on an HIV-1 patient with NSCLC [30].

### 3.4. Resistance of Savolitinib

**Figure 2:**
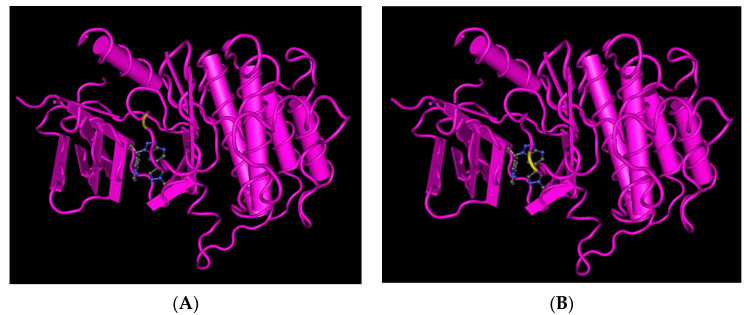
Interaction of savolitinib with the MET receptor’s residue position causes acquired resistance to savolitinib. (**A**) Position of D1228 in the MET receptor D1228H and D1228N mutations occur at this position. (**B**) Position of Y1230 in the MET receptor The Y1230C mutation occurs at this position.

Resistance to MET TKIs is inevitable. Previous studies have suggested that savolitinib resistance in NSCLC is driven in part by MYC overexpression in H1993 cells [31]. According to Melanie M. et al., the patient with gastric cancer who received savolitinib maintained a PR based on radiologic and clinical assessments. Initially, the patient’s ctDNA analysis at baseline revealed the presence of the TP53 P190L mutation with an allele frequency of 44%, along with a MET copy number of 3.0 and a MYC copy number of 5.6. In addition, new observations detected low frequencies of MET D1228H (5%), MET D1228N (5%), MET D1228V (35%), and MET Y1230C (3%). However, after 3.5 months of treatment with savolitinib, the patient experienced rapid disease progression. The patient’s ctDNA analysis revealed persistently low MET and MYC copy numbers compared to baseline. During the PR phase, the allele frequencies of MET D1228H increased to 31%, while those of MET D1228N increased to 12% compared to the ctDNA samples collected at that time. In the progression phase, the presence of MET D1228V (1%) and MET Y1230C (1%) mutations was also observed (Figure 2) [32,33,34].

A 74-year-old woman with gastric cancer and extensive liver metastases experienced rapid disease progression following TS-1 chemotherapy. She was then treated with savolitinib. Initially, ctDNA analysis during the PR phase showed no MET amplification. However, after four cycles of savolitinib, her PR was confirmed radiologically, accompanied by a slight increase in MET copy number (2.7) and the emergence of a TP53 G245D variant with a 2% allele frequency in ctDNA. The tumor size decreased by 83.5% compared to baseline. Unfortunately, after six months of treatment, radiologic progression occurred. The patient exhibited high levels of MET amplification (13 copies) and a CDK6 copy number of 3.9 at the time of disease progression. Notably, there was strong concordance between the ctDNA and tumor tissue DNA derived from liver metastasis. Both samples displayed a resurgence of MET and CDK6 amplification, which was not detected during the PR phase. Additionally, the ctDNA sample obtained at the time of progression indicated the expansion of a clone containing the TP53 G245D substitution, evidenced by an increase in allele fraction from 2% (at the third follow-up) to 22% (at progression) [32].

### 3.5. Biomarker for MET

Several studies have demonstrated the predictive value of MET biomarkers in identifying patients who will benefit most from HGF/MET-targeted therapies administered as monotherapy or in combination [35]. Inflammatory mediators, including interleukin-1-alpha (IL-1alpha), IL-1b, tumor necrosis factor-alpha, and prostaglandin E2, increase the gene expression of HGF in stromal cells. It is likely that these inflammatory mediators are involved in the upregulation of HGF in tumors because they are increased in the tumor microenvironment and contribute to a drug-resistant and/or metastatic tumor microenvironment [36]. In addition, MET gene amplification and/or protein overexpression are frequent in cancer, accelerating research on intratumoral MET gene copy number or circulating soluble DNA, intratumoral MET protein content, and phosphorylation (activation) status [37].

The VIKTORY (targeted agent eValuation In gastric cancer basket KORea) trial is the first and largest platform study in gastric cancer, supporting both the validity and clinical utility of tumor profiling. This study was designed to stratify patients with metastatic gastric cancer based on clinical sequencing and assign patients to one of 10 s-line treatment-related clinical trials, focusing on eight biomarker groups (RAS aberrations, TP53 mutation, PIK3CA mutation/amplification, MET amplification, MET overexpression, all negative, TSC2 deficiency, and RICTOR amplification). The study showed that treatment cohorts assigned to biomarkers had encouraging response rates and survival compared to conventional second-line chemotherapy. Analysis of circulating tumor (ctDNA) showed a good correlation between high MET copy number by ctDNA and response to savolitinib [38].

ctDNA biomarkers also allow longitudinal monitoring of clinical outcomes with savolitinib in patients with MET exon 14 skipping mutation-positive NSCLC and other NSCLC subtypes. Specifically, undetectable baseline MET exon 14 skipping mutations or post-treatment clearance may predict favorable clinical outcomes, and secondary MET mutations and other acquired genetic alterations may explain resistance to savolitinib [39].

## 4. Trials for Savolitinib

### 4.1. In Vivo and Xenograft Study

Savolitinib has been shown to inhibit the growth of gastric cancer cell lines in in vitro studies. Additionally, in vivo studies have demonstrated anti-tumor activity in models of gastric cancer with *MET* amplification and papillary renal cell carcinoma (PRCC) [40]. A pharmacokinetic-pharmacodynamic (PK-PD) model conducted by Jones et al. found that savolitinib effectively inhibited the activity of phospho-MET, a protein associated with cancer, in a xenograft mouse model using human lung and gastric cancer cell lines [25].

### 4.2. First in Human Phase I Trial

A phase 1 clinical trial was conducted in Australia (NCT01773018), involving 48 patients with locally advanced solid tumors. Savolitinib was administered at various doses, with a maximum tolerated dose of 800 mg. The trial showed the preliminary effectiveness of savolitinib in patients with papillary renal cell carcinoma who had MET gene copy number alterations. The most common adverse events reported were nausea (63%), vomiting (42%), fatigue (35%), and peripheral edema (27%). Savolitinib was considered tolerable, and the recommended phase 2 dose was established at 600 mg daily [41,42].

In another phase 1 clinical trial conducted in China (NCT019855), involving patients with progressive tumors and MET mutations, savolitinib demonstrated a manageable safety profile and promising anti-tumor activity. Although no partial response (PR) was achieved, there was a significant reduction in tumor size in some lesions (55% and 27%). The most common treatment-related side effects included nausea (29.4%), vomiting (27.1%), and peripheral edema (21.2%). The recommended phase 2 dose of savolitinib in this trial was set at 600 mg once daily or 500 mg twice daily. There were similarities between the patients enrolled in the phase 1 clinical studies conducted in Australia, allowing for a comparative analysis of the results [41,42].

### 4.3. Pivotal Phase 2 Trial

A pivotal Phase 2 trial (NCT02897479) was conducted in China to assess the efficacy and safety of savolitinib in patients with non-resectable or metastatic NSCLC carrying the *MET* exon 14 skipping mutations. The trial included 70 patients who received savolitinib monotherapy until disease progression or unacceptable toxicity. The daily dosage ranged from 400 mg (less than 50 kg) to 600 mg (more than 50 kg) based on patient weight. Most enrolled patients were elderly and had advanced NSCLC with prior systemic treatment.

The trial analyzed the data using the Independent Review Committee (IRC) and investigator assessments in the full analysis set (FAS) and tumor response evaluable set (TRES). Among the TRES patients evaluated by the IRC, there were 62 patients. Most patients in the FAS had an Eastern Cooperative Oncology Group (ECOG) performance status of 1. Subset analyses were performed comparing different NSCLC subtypes and treatment experiences.

With a median follow-up duration of 28.4 months, the trial demonstrated encouraging efficacy results. The median overall survival (OS) was 12.5 months, with 18-month and 24-month OS rates of 42.1% and 31.5%, respectively. Pretreated patients had a median OS of 19.4 months, while treatment-naive patients had a median OS of 10.9 months. Patients with primary squamous cell carcinoma (PSC) had a median OS of 10.6 months, while other NSCLC subtypes had a median OS of 17.3 months. Patients with brain metastases had a median OS of 17.7 months. No new safety concerns were observed with prolonged follow-up and exposure, indicating that savolitinib had an acceptable safety profile [43,44,45].

### 4.4. Phase 3 Trials on the Way

Currently, there are four phase 3 clinical trials investigating the efficacy and safety of savolitinib in various treatment settings for NSCLC patients.

Phase 3b Study (CTR20211151) aims to evaluate the efficacy and safety of savolitinib in two cohorts of locally progressive or metastatic NSCLC patients with MET exon 14 skipping mutations in China. One cohort includes patients who have previously received platinum-based chemotherapy but have experienced disease progression or intolerable toxicity, while the other cohort consists of patients who have not received systemic chemotherapy for advanced disease. Patients in both cohorts are treated with savolitinib until disease progression or intolerable toxicity.

Phase 3 Clinical Trial SACHI (CTR20211441), which is a randomized, two-arm, open-label, multi-organ study, is being conducted in China. It aims to evaluate the efficacy and safety of savolitinib in combination with osimertinib (an EGFR-TKI) compared to chemotherapy in Chinese patients with MET-amplified NSCLC who developed the disease after treatment with 1st to 3rd generation EGFR-TKIs. This trial has already started recruiting patients at multiple centers.

SAFPRON Phase 3 Clinical Trial focuses on advanced NSCLC patients worldwide with advanced MET amplification or MET overexpression after treatment with osimertinib. It aims to evaluate the efficacy and safety of the combination therapy of savolitinib plus osimertinib compared to chemotherapy.

SANOVO Phase 3 Clinical Study (NCT05009836) is evaluating the efficacy and safety of the combination therapy of savolitinib and osimertinib in patients with EGFR mutation-positive NSCLC and MET overexpression who have not received prior treatment.

## 5. Non-Small Cell Lung Cancer (NSCLC)

### 5.1. Non-Small Cell Lung Cancer

Non-small cell lung cancer (NSCLC) is the most common type of lung cancer, accounting for approximately 85% of all lung cancers. It has considerable heterogeneity and may be associated with some known and/or unknown causative gene changes. NSCLC has a very poor prognosis, with significantly lower OS and 5-year survival rates compared to other types of lung cancer.

NSCLC is increasingly treated with targeted therapies. Savolitinib is a highly selective MET-TKI for advanced NSCLC with a *MET* exon 14 skipping mutation. *MET* exon 14 skipping mutation is most common in NSCLC. Mutations resulting in the loss of exon 14 in the *MET* gene lead to dysregulation and inappropriate signaling, which are associated with increased responsiveness to MET TKIs [46,47].

*MET* amplification is indeed more common in *MET* exon 14 skipping mutation NSCLC, with reported frequencies of 15–21%. *MET* amplification occurs about 4% in lung adenocarcinomas and 1% in squamous cell lung cancer. The overall incidence of *MET* exon 14 skipping mutations in NSCLC is 3% to 4% with untreated NSCLC or previously treated molecularly driven NSCLC, such as EGFR, and acts as a mechanism of acquired resistance. Adenocarcinoma has a relatively higher incidence than squamous cell lung cancer (1.5–2.0%). Pulmonary sarcomatoid carcinoma has 20–30% *MET* exon 14 skipping mutations [48]. The *MET* exon 14 skipping mutations itself occurs with higher frequency in Caucasian patients compared to Asian patients, and it is more frequently observed in smokers or ex-smokers. However, it is worth noting that approximately one-third of patients with *MET* exon 14 skipping mutation NSCLC are never smokers. Furthermore, the *MET* exon 14 skipping mutations is more commonly found in women than in men. In general, *MET* exon 14 skipping mutation NSCLC tends to occur in older individuals, with a median age of 74, which is higher than NSCLC cases with other molecular drivers [49]. Data from retrospective studies have suggested attenuated activity of immune checkpoint inhibitors in patients with *MET* exon 14 skipping mutations, independent of expression of programmed death ligand-1 (PD-L1) [50].

Savolitinib has demonstrated efficacy in first- and second-line settings, including in patients with NSCLC and aggressive pulmonary sarcomatoid carcinoma, and has an acceptable safety profile. It can also cross the blood-brain barrier and has demonstrated some activity against central nervous system metastases [45]. Patients with exon 14-skipping NSCLC had higher response rates than patients with overexpressed or amplified MET protein and had higher rates of intracranial disease control. Therefore, savolitinib may become a new standard of care to address MET dysregulation in patients with advanced or metastatic NSCLC, even those with brain metastases [51,52].

### 5.2. Neoadjuvant Therapy in NSCLC

Savolitinib has been shown to be effective in neoadjuvant chemotherapy for lung cancer, specifically in cases of *MET* exon 14 skipping mutation-positive locally advanced primary lung adenocarcinoma. In one case, a patient received 5 weeks of neoadjuvant treatment with savolitinib and experienced a significant reduction in tumor burden and lymph node size. Subsequently, a successful lobectomy and lymph node dissection were performed, resulting in a pathological response of 50% and a post-operative pathological staging of pT1cN0M0, IA3 [53].

In another study, three patients with locally advanced, unresectable NSCLC received induction therapy with savolitinib as a first- or second-line treatment after their disease had progressed following pre-operative chemotherapy. All three patients experienced a significant reduction in tumor size, with previously unresectable tumors becoming resectable after treatment with savolitinib [54]. In a separate case, a 76-year-old male patient with resectable stage IIIB lung adenocarcinoma harboring the *MET* exon 14 skipping mutations was successfully treated with savolitinib. After neoadjuvant savolitinib, the primary tumor shrank by 82%, and only 5% of the tumor remained viable at the time of subsequent radical surgery [55,56].

Another case report described a patient with the *MET* exon 14 skipping mutation in NSCLC who was initially unsure of intrapulmonary metastases and had recently undergone percutaneous coronary intervention for acute myocardial infarction. The patient received savolitinib at a dose of 600 mg once daily and experienced significant tumor shrinkage. After six months, no metastatic lesions were found, and the patient was diagnosed with early-stage lung cancer. A radical tumor resection was performed, and the patient recovered successfully [57].

Based on these cases, savolitinib appears to be a valuable treatment strategy for patients with *MET* exon 14 skipping mutation NSCLC, particularly those who are not suitable candidates for surgery. It has demonstrated the potential to reduce tumor size, make previously unresectable tumors resectable, and improve pathological responses in the neoadjuvant setting.

### 5.3. Lung Sarcomatoid Carcinoma

Pulmonary sarcomatoid carcinoma (PSC) is a rare and aggressive subtype of NSCLC that accounts for a small percentage of primary lung cancers. PSC is characterized by poor differentiation and more aggressive behavior compared to other types of lung cancer. Survival rates for PSC are generally low, and patients with advanced disease have poor outcomes, including survival rates of less than 5% and shorter survival times [48,58].

Recent studies have focused on gene mutations associated with PSC, particularly in the *MET* proto-oncogene. The most common and well-studied mutation is the exon 14 skipping mutation, which is found in a significant proportion of PSC cases. In addition, amplification and overexpression of the *MET* gene have been observed in a smaller percentage of patients. These molecular alterations have important implications for targeted therapies in PSC [48].

A Phase 2 clinical trial is underway to evaluate a treatment specifically targeting the *MET* exon 14 skipping mutations in patients with locally advanced or metastatic NSCLC, including those with sarcomatoid histology. Preliminary data from the trial, involving 70 patients, demonstrated promising results. The treatment showed a notable ORR of 47.5% and a median progression-free survival of 6.8 months. Importantly, these positive outcomes were observed in patients who had not received prior MET inhibitor therapy.

### 5.4. Combination Therapy for NSCLC

There have been studies in which MET TKI (especially in NSCLC with a *MET* exon 14 skip mutation) can be seen as a combination therapy with EGFR TKI [59].

- savolitinib + osimertinib

The combination of savolitinib and osimertinib has shown promising results in preclinical studies and clinical trials for the treatment of NSCLC with EGFR mutation, *MET* amplification, or *MET* overexpression.

In preclinical models, the combination of savolitinib and erlotinib (a first-generation EGFR-TKI) demonstrated significant tumor suppressive effects in NSCLC cell line models with *MET* amplification. The combination of savolitinib and osimertinib showed superior anti-tumor activity compared to monotherapy in NSCLC models with EGFR mutations and *MET* amplification [60].

Phase 1 Study (TATTON, Part A): The combination of savolitinib and osimertinib was evaluated in patients with advanced NSCLC who had previously progressed on EGFR-TKI treatment. The study demonstrated the safety and resistance of Osimertinib and Savolitinib in patients with advanced NSCLC (*n* = 18) who had previously advanced disease after EGFR-TKI treatment [61,62]. The dose of savolitinib was increased from OD 600 mg to 800 mg, with Osimertinib 80 mg as a fixed dose. The study demonstrated promising anti-tumor activity with an objective response rate (ORR) of 44%. Extended cohorts of the TATTON study evaluated the combination of savolitinib and osimertinib in patients with *MET*-amplified and EGFR mutant-positive NSCLC. The ORR was 49% in patients with *MET* amplification and an EGFR mutation. The combination therapy showed a manageable safety profile [63].

Phase 2 Study (SAVANNAH) evaluated the efficacy of savolitinib in combination with osimertinib in patients with *EGFR* mutation, *MET* amplification, and/or *MET* overexpression. The overall response rate (ORR) was 32%, and the median duration of response was 8.3 months. Patients with high *MET* amplification or high threshold *MET* overexpression showed better efficacy [64].

The FLOWERS trial is investigating the efficacy and safety of osimertinib in patients with novel *MET* amplification and/or *MET* overexpression, with or without savolitinib. The SAFFRON trial is comparing the combination of savolitinib and osimertinib with platinum-based chemotherapy in patients with NSCLC after osimertinib treatment [65].

A phase 3 trial, the SAFFRON trial (NCT05261399), is underway to compare savolitinib and Osimertinib combination therapy with platinum-based double chemotherapy in patients with disease-induced NSCLC (*EGFR* mutation, *MET* overexpression, and/or *MET* gene amplification) after Osimertinib treatment.

Overall, the combination of savolitinib and osimertinib has shown promising anti-tumor activity and manageable safety profiles in patients with EGFR mutation, *MET* amplification, or *MET* overexpression in NSCLC.

B. savolitinib + gefatinib

A phase 1 clinical study (NCT02374645) conducted in China evaluated the combination of savolitinib and gefitinib (a first-generation EGFR-TKI) in patients with advanced disease EGFRm and *MET* amplification in NSCLC. The study demonstrated promising anti-tumor activity. Two different doses of savolitinib, 600 milligrams and 800 milligrams, were administered along with 250 milligrams of gefitinib. The ORR for patients with EGFR T790M negative and positive status was 52% and 9%, respectively. Overall, the daily combination of savolitinib 600 mg and gefitinib 250 mg showed acceptable safety profiles and promising anti-tumor activity in patients with advanced EGFRm and *MET* amplification NSCLC who had received previous EGFR-TKI treatment [66].

C. savolitinib + durvalumab

The ongoing SOUND Clinical Trial (NCT05374603) is an open-label, multicenter, exploratory clinical trial that aims to investigate the combination therapy of savolitinib and durvalumab in Chinese patients with EGFR wild-type topical or metastatic NSCLC who have *MET* mutations. The trial will include 30 patients with *MET* amplification and 30 patients with *MET* exon 14 skipping mutations. These patients will receive treatment with 1500 mg of durvalumab and 300 to 600 mg of savolitinib (once daily) for a 28-day treatment cycle. The treatment will continue until disease progression, death, or the occurrence of toxicity. The trial is currently ongoing.

## 6. Renal Cell Carcinoma

Papillary renal cell carcinoma (PRCC) is the most common subtype of non-clear cell renal cell carcinoma and has a poor prognosis, particularly in advanced stages [40]. Since some cases of PRCC are driven by the *MET* gene, targeting MET may be a promising therapeutic approach. Savolitinib has demonstrated anti-tumor activity in patients with PRCC. In previous studies, *MET*-driven PRCC has shown better treatment response compared to *MET*-independent PRCC [67,68].

The SAVOIR phase 3 clinical trial, a multicenter study, evaluated the efficacy of savolitinib compared to sunitinib, a standard treatment for advanced PRCC. The trial enrolled 60 patients, with most having chromosome 7 gain and no prior therapy. However, due to the availability of external data on progression-free survival (PFS) with sunitinib in patients with MET-driven disease, enrollment in the study was closed prematurely. Preliminary results showed that savolitinib had numerically greater median PFS, OS, and ORR compared to sunitinib. However, the difference in median PFS between the two groups (7.0 months for savolitinib and 5.6 months for sunitinib) was not statistically significant. Grade 3 or higher adverse events (AEs) were less frequent in the savolitinib group compared to the sunitinib group, and fewer dose modifications related to AEs were required. After discontinuation of treatment, a higher proportion of patients in the savolitinib group received subsequent anticancer therapy. Although the study had limited patient numbers and follow-up, savolitinib demonstrated promising efficacy and a more favorable safety profile compared to sunitinib in MET-driven PRCC. Further investigation is needed to determine the potential of savolitinib as a treatment option for MET-driven PRCC [68].

The combination therapy of savolitinib and durvalumab showed remarkable effectiveness in overcoming drug resistance and demonstrated a high clinical response rate in exploratory MET-based metastatic papillary renal cell cancer. Durvalumab has side effects such as rash (48%), vomiting (43%), and diarrhea (39%) [69].

## 7. Gastric Cancer

The phase 2 VIKTORY umbrella trial showed that in patients with metastatic and/or recurrent gastric adenocarcinoma, savolitinib monotherapy had an ORR of 50% (10/20) in the subset of gastric cancer patients with *MET* amplification. Further genomic analysis showed that patients with a *MET* GCN > 10 by tissue next-generation sequencing had an ORR of 70% for savolitinib, inferring that the subset of patients with *MET* amplification experienced a greater absolute reduction in tumor burden [38].

A case was reported of a 35-year-old man with advanced gastric cancer and bone, adrenal, and lumbar-2 vertebral metastases. The patient was resistant to chemotherapy, in poor general condition, and had thrombocytopenia and anemia. NGS analysis revealed *MET* gene amplification in the tumor. After 39 days of daily treatment with 400 mg savolitinib, the patient achieved a PR, and both anemia and thrombocytopenia improved. No significant side effects were observed, and the patient remained progression-free for 14 weeks [70].

Another case showed that a 31-year-old woman underwent a total gastrectomy in 2013 for stage pT4N3M0 gastric cancer. The tumor was a poorly differentiated tubular adenocarcinoma, and the patient was microsatellite stable and HER2-negative. Oophorectomy revealed MET IHC3+ and *MET* amplification confirmed by FISH. With savolitinib treatment, the patient experienced a significant reduction in tumor volume, achieving a PR lasting six months. The maximum tumor diameter decreased by 47.7% compared to the baseline measurement. Genomic sequencing of ctDNA samples indicated that the patient had a non-shedding tumor, as no variants, including *MET* amplification or mutations, were detected across a 100-gene panel [32].

The other case presented was a case study of a 47-year-old male with advanced gastric cancer, bone marrow invasion, and extensive metastases. The patient experienced severe pain, thrombocytopenia, and hemorrhagic anemia. Due to chemotherapy resistance, the patient underwent monotherapy with savolitinib. Savolitinib was administered based on the presence of *MET* gene amplification and rearrangement in the tumor. Following savolitinib treatment, the patient’s condition improved significantly, achieving partial remission. At the time of reporting, the patient had remained alive and free of disease progression for 15 weeks without any notable adverse reactions. Additionally, another female gastric cancer patient with *MET* amplification who received savolitinib monotherapy as a third-line treatment also showed no signs of disease progression for 12 weeks [71].

In a phase I trial, it was suggested that the combination of savolitinib at a dose of 600 mg once daily with docetaxel at a dose of 60 mg/m^2^ would be the recommended phase II dose. This combination therapy showed highly encouraging anti-tumor effects and resulted in sustained responses among patients with *MET*-amplified Gastric cancer in the subsequent phase II clinical trial [72].

## 8. Hepatocellular Carcinoma

Systemic treatment of hepatocellular carcinoma (HCC) includes immune checkpoint inhibitors, bevacizumab, and other TKIs. It is not the primary option of choice due to poor overall response and median progression-free survival. A case has been reported of a patient with *MET*-amplified HCC progressing after 3 months on bevacizumab and sintilimab and maintaining PR for more than 8 months with manageable adverse events with savolitinib. This suggests that savolitinib may be a therapeutic option for *MET*-amplified HCC [73].

## 9. Colorectal Cancer

The phase II study (NCT03592641) is currently underway to determine how well savolitinib works in treating patients with *MET*-amplified inoperable colorectal cancer. They have RAS-wild-type mCRC and have previously been treated with standard therapies. *MET* amplification will be detected using a blood-based genomic profiling assay. Savolitinib may inhibit the growth of tumor cells by blocking some of the enzymes needed for cell growth. Patients receive oral savolitinib 600 mg daily on days 1–28. Cycles are repeated every 28 days in the absence of disease progression or unacceptable toxicity. The study aims to estimate the ORR of savolitinib in this patient population. Secondary objectives include evaluating progression-free survival, duration of response, safety, and tolerability. The study will also explore the correlation between tissue- and blood-based biomarkers and clinical outcomes. Blood samples will be collected at baseline and during restaging to determine if savolitinib eliminates *MET* amplification in circulating cfDNA [74].

## 10. Pancreatic Cancer

Pancreatic cancer, specifically pancreatic ductal adenocarcinoma (PDAC), is a highly aggressive illness known for its tendency to spread early. It is characterized by a dense and collagen-rich supportive tissue called desmoplasia or stroma, which is primarily generated by pancreatic stellate cells (PSCs). PSCs have a role in communicating with cancer cells and other stromal cells, thereby facilitating the advancement of the disease. One specific pathway involving growth factors that potentially enables this interaction is the hepatocyte growth factor (HGF)/MET pathway. HGF is produced by PSCs, while its receptor MET is present in pancreatic cancer cells and endothelial cells [54]. The activation of the MET/HGF pathway is a consequence of the tumor microenvironment (TME) that supports tumor growth. The TME is a significant source of HGF, and MET/HGF signaling influences the TME by directly affecting stromal cells expressing *MET*. Therefore, targeting the MET/HGF pathway could be an option for adjuvant therapy in pancreatic cancer. One of the reasons for chemotherapy resistance in PDAC is the extensive desmoplastic reaction surrounding the tumor, which creates a physical barrier that hinders drug penetration. In this context, reducing the metastatic potential of cancer cells and reprogramming the dysfunctional tumor microenvironment could have potential benefits for the treatment of pancreatic cancer.

Studies of other MET TKIs such as cabozantinib, crizotinib, and capamatinib for pancreatic cancer have shown that studies suggest that TKIs such as savolitinib may be effective in pancreatic cancer. In 2013, Hage et al. examined the therapeutic potential of cabozatinib through in vitro studies. They observed that the effectiveness of gemcitabine, a commonly used chemotherapy drug, was enhanced even in pancreatic cancer (PC) cells that had developed resistance to high levels of gemcitabine. Additionally, they investigated patient-derived primary spheroidal cultures that were enriched in cancer stem cell markers and found that the agent demonstrated increased efficacy in these cultures as well [75].

In vitro experiments showed a synergistic interaction between crizotinib and gemcitabine, as evidenced by the reduced growth of primary PDAC cells. Similarly, in vivo experiments revealed a synergistic effect on primary tumor growth. However, the impact of this combination on metastatic spread remains unclear and requires further investigation [76].

In an in vivo study utilizing mouse models of PC, treatment with capamatinib demonstrated a reduction in the movement of PC cells. The treatment group exhibited a 30% lymph node involvement, whereas the control group had a higher 60% involvement, indicating a potential suppression of metastasis. Furthermore, the researchers investigated various PC cell lines (human and murine) to confirm *MET* expression and evaluate the cells’ response to capamatinib in the future. They observed that inhibiting MET decreased the proliferation and migration of PC cells induced by hepatocyte growth factor (HGF) [77].

Based on this, we believe that savolitinib, a MET TKI targeting the MET/HGF pathway, may be beneficial in the treatment of pancreatic cancer, and further studies may be conducted in the future.

## 11. Conclusions

With the development of NGS technology, MET TKIs are being actively studied in addition to traditional targeting agents. Particularly, savolitinib, a small molecule, highly selective type Ib MET TKI, is being developed for the treatment of non-small cell lung cancer with *MET* mutations. It has been developed for the treatment of non-small cell lung cancer, breast cancer, head and neck cancer, colorectal cancer, gastric cancer, pancreatic cancer, and other gastrointestinal cancers and has demonstrated good efficacy in preclinical and clinical studies.

In non-small cell lung cancer, savolitinib has shown promising anti-cancer activity in chemotherapy-resistant patients with single or combination therapy and has also shown promise in kidney, gastric, liver, and pancreatic cancers. Through interactions with the HGF/MET signaling pathway in the tumor microenvironment, we can expect savolitinib to be particularly effective in pancreatic cancer. The results of these preclinical studies are encouraging for further research, and savolitinib is expected to be a promising treatment for other types of cancer, including gastrointestinal cancers, where *MET* mutations are found.
